# Drying in a microfluidic chip: experiments and simulations

**DOI:** 10.1038/s41598-017-15718-6

**Published:** 2017-11-14

**Authors:** Paolo Fantinel, Oshri Borgman, Ran Holtzman, Lucas Goehring

**Affiliations:** 1 Max Planck Institute for Dynamics and Self-Organization (MPIDS), Göttingen, 37077 Germany; 2 Department of Soil and Water Sciences, The Hebrew University of Jerusalem, Rehovot, Israel; 3 School of Science and Technology, Nottingham Trent University, Nottingham, NG11 8NS UK

## Abstract

We present an experimental micro-model of drying porous media, based on microfluidic cells made of arrays of pillars on a regular grid, and complement these experiments with a matching two-dimensional pore-network model of drying. Disorder, or small-scale heterogeneity, was introduced into the cells by randomly varying the radii of the pillars. The microfluidic chips were filled with a volatile oil and then dried horizontally, such that gravitational effects were excluded. The experimental and simulated drying rates and patterns were then compared in detail, for various levels of disorder. The geometrical features were reproduced well, although the model under-predicted the formation of trapped clusters of drying fluid. Reproducing drying rates proved to be more challenging, but improved if the additional trapped clusters were added to the model. The methods reported can be adapted to a wide range of multi-phase flow problems, and allow for the rapid development of high-precision micro-models containing tens of thousands of individual elements.

## Introduction

The drying of porous materials is an important example of a two-phase flow process that occurs in both natural and engineered systems^1^; it is involved in soil-atmosphere energy and moisture exchange^2^, in solute transport within soils^3^ and in agriculture^4^. In such situations an invading fluid (air) displaces a more viscous defending fluid, as the latter evaporates^5^. As drying proceeds, periods of evaporation from a relatively static air-liquid interface are interrupted by sudden invasion events (Haines jumps)^6,7^. When such jumps, or bursts, occur, these interfaces depin and advance to their next stable position. This motion induces liquid flows that can cause connected interfaces elsewhere to readjust, often very rapidly^7,8^.

Drying in porous materials can be separated into two different stages, based on their characteristic drying rates and transport mechanisms. During the first stage liquid is transported mostly *via* flow from the bulk, through connected liquid pathways, to a surface where evaporation occurs^9^. During this time the evaporation rate is fairly constant (constant rate period) and influenced mostly by surface wetness, the size distribution of surface pores and the surface boundary conditions, *e*.*g*. wind^10–12^. Stage two, or the falling rate period, is identified with the loss of connectivity between the exposed surface and the liquid in the pore-space. As the fluid interface recedes into the porous medium, relatively slow vapour diffusion becomes the dominant transport mechanism^13,14^ causing the drying rate to drop noticeably.

Experimentally, progress in understanding the basic physics of transport in porous media has been made in different ways including the use of etched channel networks^15–17^, Hele-Shaw cells containing solid pillars^18,19^ or monolayers of silica spheres^5,20^. These experiments have typically focused on long throat networks^15–17^, scales that are significantly larger than real soils^18^ or pore networks with a few key features^21,22^, due to the challenges of solving manufacturing problems that arise when precisely crafting many micron-sized fluid channels.

Computationally, modelling a three dimensional porous system is also still a difficult task, although many attempts have been made in this direction. This is due to the need to limit numerical resolution when dealing with large numbers of variables^18^. A possible solution is to approach the problem from a two-dimensional description and, once the main features are accurately captured, expand the model into three-dimensions. Pore-network models (PNM), originally proposed by Fatt *et al*.^23^, have become accepted in modelling multi-phase flow in porous media^24^, thanks to their combination of computational efficiency and the ability to capture the essential pore-scale physics, while coarse graining over sub-pore effects. For example, a porous medium may be represented as a collection of simple objects like pores and throats, which interact according to different physical mechanisms such as capillarity, gravity, pressure dissipation by flow, *etc*. Pore-network modelling has been used to capture different pore-scale physical processes including drainage, imbibition, solute transport, biofilm growth, reactive dissolution and precipitation, and more^25^. Early applications to drying were made by Nowicki *et al*.^26^ and Prat^27^. They were able to calculate effective permeabilities^26^ and to estimate the stabilising effects of gravity on the invasion front into a drying body^27^. Generally, PNM of drying arrays of pores and throats are based on mass balances of liquid and vapour in single pores: transport can occur through diffusion of vapour and viscous liquid flow^28^. PNM have also shown the effects of capillary pumping on invasion patterns^29^ and how liquid flow through films or corners modify drying rates^30,31^. Furthermore, PNM can be coupled with an external, diffusive, boundary layer, allowing for solutions of problems involving complex boundary conditions and processes^16^. The strength of PNM lies in their ability to capture complex effects efficiently, allowing one to investigate the minimum assumptions needed to accurately describe, or predict, observational results.

**Figure 1:**
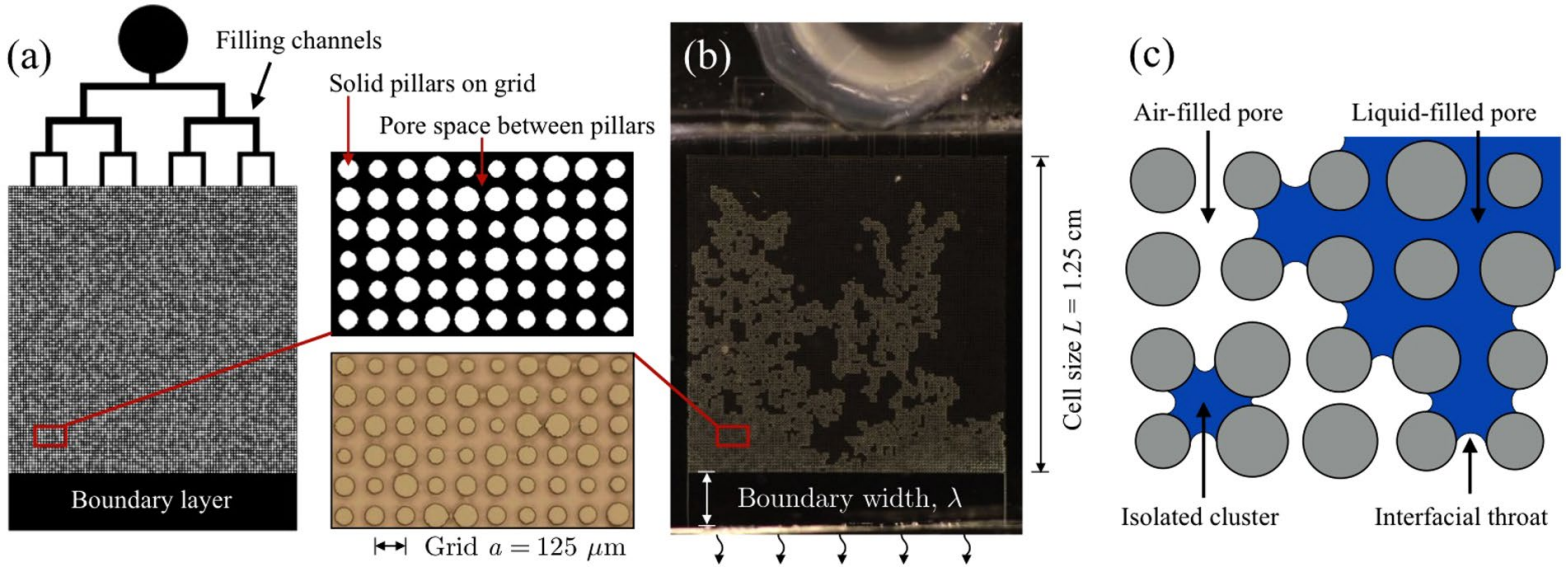
From design to experiment and simulation. The mask design (**a**) shows how a microfluidic chip is conceived, with a pore space containing several thousand microscopic pillars. These pillars define a regular grid of pores, with randomised throats separating neighbours. An inlet and channels allow for the uniform filling of a volatile fluid into the pore space. A chip (**b**) reproduces the design and is sealed on all sides, except at the edge of a boundary layer, where evaporation can occur. The pore space in the chip is 40 *μ*m thick, and the magnified regions compare some designed and realised features. (**c**) A pore-network model is developed with the same geometry, with pillars on a grid. The pores can be either liquid-filled (here, blue) or air-filled (white). Any two adjacent pores are connected *via* a throat, which can trap fluid-air interfaces. Each connected cluster of wet pores has a capillary pressure *p*, that is related to the curvature of menisci in throats along its surface. The experimental and simulated drying patterns, resulting from identical geometries, can thus be compared.

In this paper we aim to connect pore-scale observations of drying phenomena in random porous media with their macroscopic interpretation by developing a type of experimental micro-model that is based on microfluidic techniques, as shown in Fig. 1. This method can manufacture large two-dimensional porous systems with complete control of features at a scale of a few micrometers, a length-scale similar to many natural pore-scales, and with system sizes large enough to systematically resolve heterogeneities. As such, we include here a detailed study of the reproducibility of such experiments, and the tolerance expected for studies of multi-phase flow patterns. We also compare and contrast these micro-models with a complimentary PNM. Briefly, we find that such a model accurately captures the dynamics and pattern of the leading front of the drying, but disagrees on the formation and evolution of trapped clusters of fluid.

Our manufacturing, experimental and numerical techniques are detailed in the Methods section at the end of this manuscript, and outlined in Fig. 1. A companion paper^32^ further explores the effects of spatially correlated disorder on drying.

## Results

We have used soft lithography methods^33–36^ that allow for precise control over the positioning of micron-scale objects to make microfluidic chips containing a pseudo-two-dimensional granular medium. Specifically, we make chips containing a regular 100 × 100 array of solid pillars, with a grid spacing *a* = 125 *μm*, extending through a Hele-Shaw cell 40 *μ*m thick. We have also imposed a heterogeneity on the size distribution of the grains and pores, in order to investigate the effects of disorder on drying behaviour in porous media. The microfluidic chips, or cells, were filled with a volatile oil and allowed to dry horizontally (*i*.*e*. without gravitational effects). Evaporation proceeds through one open edge that is separated from the porous medium by a stagnant boundary layer of 1–3 mm width, which is free of pillars but maintains the same thickness as the rest of the pore-space. The experiments were then compared to pore-network model simulations, of the same geometries.

In the following we start by presenting some typical experiments, and use these to explore drying rates and to define the metrics used in this study. We then proceed to test how reproducible our microfluidic experiments are and the corresponding sensitivity of our model. Finally, we compare the finer details of the patterns observed in experiments to those observed in the model, in order to observe the effects of pore-scale disorder on drying and to assess the successes and limitations of the pore-network approach, with a view for future applications.

### Drying rates and non-dimensionalisation

**Figure 2:**
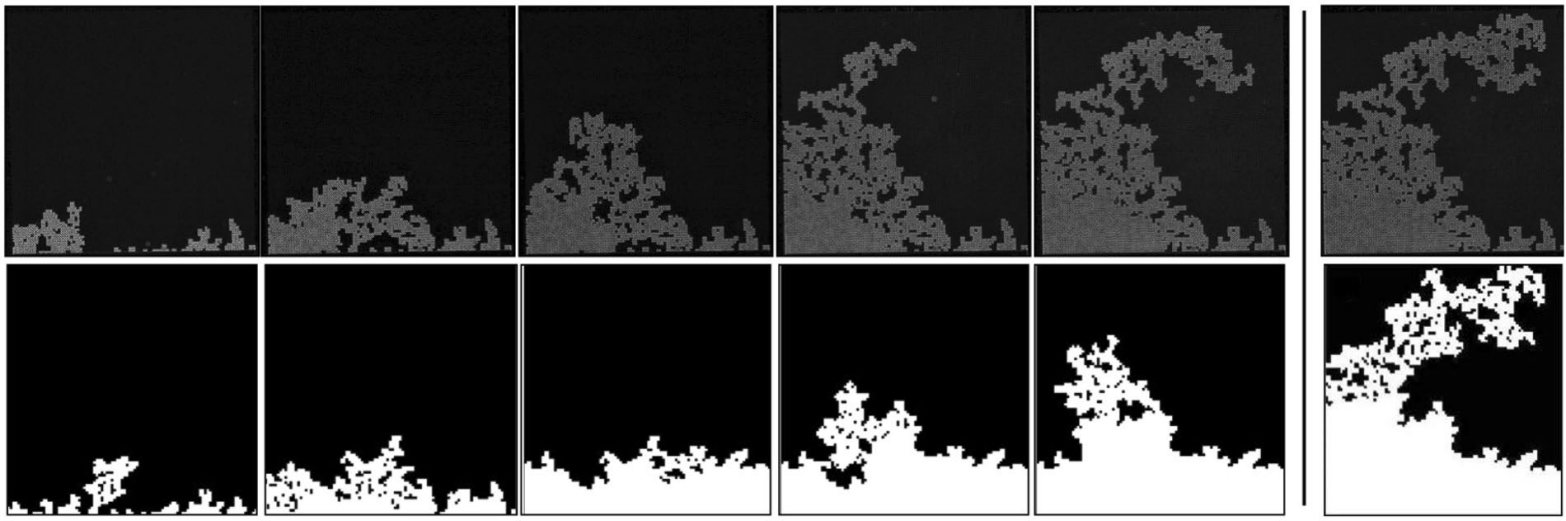
Comparison between experimental (top) and simulated (bottom) drying patterns using identical pore geometries, for a chip with 20% random disorder in pillar size. The time step between experimental images is 200 min, and snapshots of the simulation are given at corresponding total liquid saturations. The last panels compare the patterns at breakthrough. Here, and throughout this paper, the area invaded by air is shown in white, while the fluid-saturated region is shown as black.

An experiment begins with the onset of air invasion into the drying porous medium, and a representative invasion sequence is sketched in Fig. 2. In all experiments we observe an initial phase where the drying rate, $\dot{E}$ (measured as a volumetric flux; typical value 0.5 *μ*m/s), is constant to within experimental accuracy. The drying rate in this constant rate period depends on the size of the boundary layer, *λ*, as for early drying the pore geometry of the cell is expected to have little to no effect on the drying dynamics^10^. In some cases the evaporation rate remains relatively stable until breakthrough, *i*.*e*. when the drying front reaches the filling end of the cell. During the constant rate period the measured $\dot{E}$ still fluctuates with a standard deviation of ~0.1 *μ*m *s* ^−1^ around the mean rate, in any particular experiment. We take this as our absolute measurement uncertainty.

In the initial situation of a saturated pore-space, the potential drying rate can be predicted from Fick’s laws to be ${\dot{E}}_{0}=D{P}_{sat}m/\lambda RT{\rho }_{liq}$, where *P*_*sat*_ = 2.1 × 10^3^ Pa is the saturation pressure of our oil, *ρ*_*liq*_ = 1614 kg/m^3^ is its liquid density, *m* = 0.414 kg/mol is its molar mass, *R* is the ideal gas constant and *T* = 298 K is the lab temperature^37^. In our pore-network model, we confirmed that the initial drying rate reduces to this situation in the limit of small pores and a thick boundary layer ($\lambda /a\gg 1$).

When compared to experiments, the simulations capture the right order of magnitude of ${\dot{E}}_{0}$, although experiments typically show a lower initial drying rate (about half of that expected). This discrepancy between the pore-network model and experiment can be reduced if we consider an additional effective boundary layer of about 3–8 mm. The difference could thus be accounted for by the existence of a layer of stagnant air outside of the microfluidic chip, with additional (and unmodelled) gradients in the vapour concentration there. Similar diffusive boundary layers are characteristic of drying in an open environment^38,39^, and would represent a thin layer of still air in the room, around the drying chip.

As we will show in the next sections, however, uncertainties in the effective boundary layer width affect the initial drying rates alone and will not significantly change the patterns formed during drying. Therefore, we use these initial drying rates to non-dimensionalise our observations. To do so, we first estimate the evaporation rate at all times by computing the numerical derivative $\dot{E}={\rm{\Delta }}A/L{\rm{\Delta }}t$, where Δ*A* is the increase in dry area from one picture in the drying sequence to the next, Δ*t* is the time interval between the two pictures and *L* is the length of the side of the cell. As this numerical differentiation introduces noise, the resulting rates are passed through a low-pass filter, with a cutoff of 10 steps, and then a 15-point moving average. The initial drying rate, ${\dot{E}}_{0}$, is also taken to be the slope of a linear fit of the evaporation rate data (*i*.*e*. of dry area *vs*. time) over the first 40 minutes of any experiment, to minimise noise. The relative drying rate is then ${E}^{\ast }=\dot{E}/{\dot{E}}_{0}$.

Finally, we define a characteristic length-scale for our experiment as the grid spacing of the pillars, *a*. For example, the sides of our cells have length *L* = 100*a*, and so a dimensionless size of L* = 100. The initial drying rate is then used to define a characteristic timescale, *τ*, as the time it would take to dry the first row of pores at a constant rate:

1 $$\tau =\frac{a}{{\dot{E}}_{0}}\to {t}^{\ast }=\frac{t}{\tau }.$$

If the drying rate was constant throughout the experiment, then t* = 100 would be the time needed to dry the whole cell.

### Minkowski functionals

**Figure 3:**
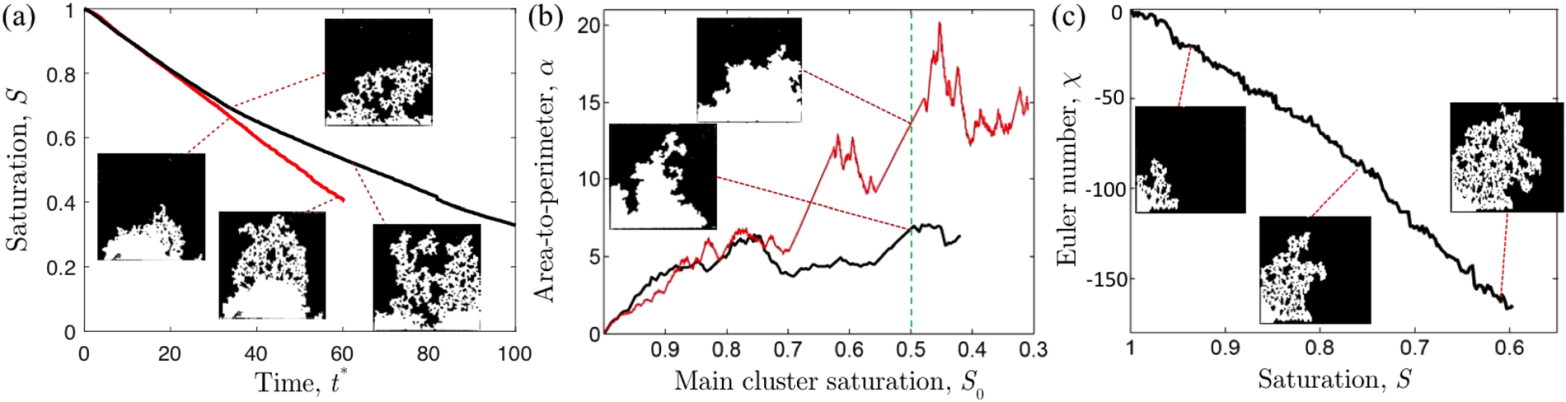
Examples of the Minkowski functionals. (**a**) The saturation *S* measures the remaining fraction of filled pore space. The red line here shows an experiment with only a constant rate period, while the black line shows a different example where the evaporation rate slows during air invasion. (**b**) The area to perimeter ratio, *α*, describes the evolution of the leading drying front (*i*.*e*. ignoring trapped clusters) in time. For a given invaded area, a smaller *α* means a higher perimeter and a rougher front: for example, the black line shows an experiment that develops a rougher perimeter than that described by the red line. Whenever a cluster is cut off from the main cluster, both *α* and *S*_0_ will jump to new values (here, shown by straight line segments). (**c**) The Euler number, *χ*, is the number of connected objects (white areas) minus the number of liquid clusters (black). A low *χ* implies many isolated clusters.

We quantitatively compare experimental and simulated drying patterns *via* the Minkowski functionals^40^. These metrics can be used to characterise all kinds of complex patterns arising, for example, from dewetting phenomena or fracture^41,42^. In two-dimensional systems, three functionals are needed: (*i*) the ratio of one phase to the total area available, *e*.*g*. the liquid saturation, *S*; (*ii*) the ratio, *α*, of this area to its perimeter and (*iii*) the Euler number, *χ*. This last metric is related to the topology of the pattern, and gives the difference between the number of connected regions and the number of holes within them. These three metrics can thus give us information about the filling state of the system at a given time (*S*) the roughness of the drying front (*α*) and the connectivity of the fluid phase (*χ*). These functionals are demonstrated in Fig. 3.

In our processed images the saturation, *S*, is the fraction of the total area covered by the liquid phase divided by the total area of the porous medium (*i*.*e*. excluding the boundary layer and filling channels). We also calculate the corresponding main cluster saturation, *S*_0_, where the main cluster is defined as all the wet pores that remain connected with the back of the cell.

To calculate the area-to-perimeter ratio we focus on the shape of the leading front, which is defined by the interface of the main cluster. The leading front has a perimeter length *P*_0_ and divides the cell between the main, fully wet cluster, and a region of area *A*_0_, which is either dry or contains isolated clusters of fluid. Its area-to-perimeter ratio, *α*, can be scaled to be non-dimensional using the characteristic grid size, *a*, as *α* = *A*_0_/*aP*_0_. A high *α* describes a compact front, and *vice versa*.

Finally, the Euler number *χ* is computed by counting the number of connected regions (the dry area, here) and subtracting the number of holes (all the isolated clusters). The more negative the Euler number is, the more isolated clusters are present.

### Repeatability

The work presented here is intended to serve as a benchmark for future applications of soft lithography methods in making microfluidic micro-models. We thus turn next to look at the reproducibility of the experimental methods, and the resulting drying patterns. A source of uncertainty in the experiments is the manufacturing of the chips. Therefore, we want to know: how well does the pillar size distribution in a chip match its design? Then, we estimate how much this error influences a drying pattern, in order to answer the question: will the drying metrics stay the same for different copies of the same design?

To answer the first question, we compared digital microscope pictures of our samples with the designs of their masks, as in Fig. 1. Measuring 200 pillars we found an average radius of 49.72 ± 0.18 *μ*m, compared to a designed mean size of 50 *μ*m. Thus, we have a negligible systematic error in manufacturing, of at most 0.28 ± 0.18 *μ*m. However, for individual pillars we found the measured radii to be, on average, 1.63 ± 0.20 *μ*m different from their specified design. This corresponds to a 3.2% random manufacturing error in feature size. During manufacture we also measured variations in the thickness of the pore space by means of a white light interferometer. Within each sample, as measured at the four corners, we tolerated variations in thickness of no more than 3 *μ*m. The samples were 38 *μ*m thick, on average.

**Figure 4:**
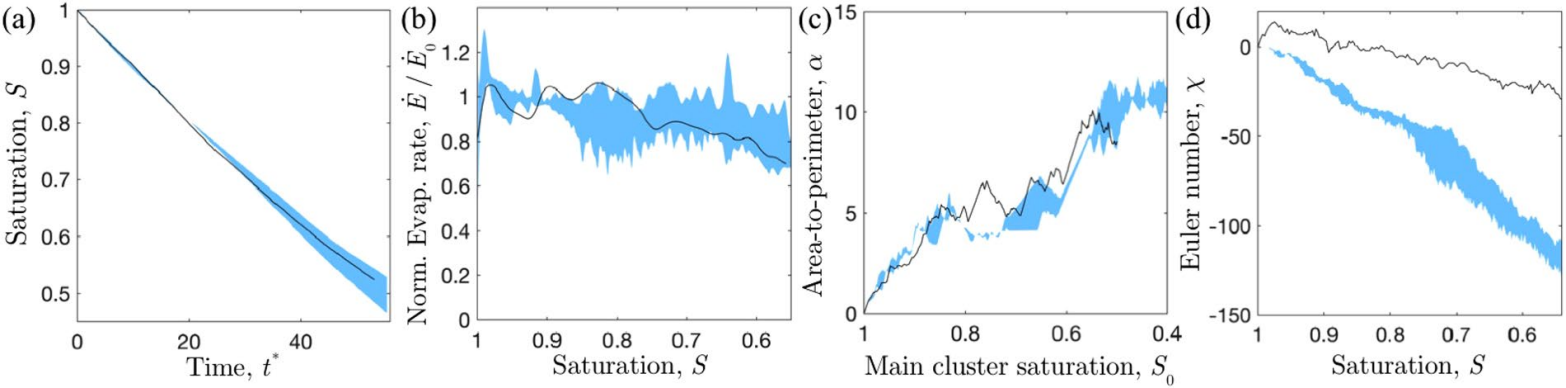
Metrics showing the experimental clouds of observations, based on three replicates of identically prepared chips (the blue area gives the mean behaviour within one standard deviation). The black lines correspond to an experiment in the same design, but with water, instead of oil, as the volatile phase. Panels show (**a**) how the fluid saturation depends on the (rescaled) time, (**b**) how the relative evaporation rate depends on fluid saturation, (**c**) how the area-to-perimeter ratio of the leading front increases with the area of the main cluster, and (**d**) the evolution of the Euler number, which characterises the number of isolated clusters left behind the leading front.

How reproducible are the drying patterns? We repeated experiments for three designs, each of which had three different chips cast from the same master. We measured the Minkowski functionals during drying in each case and estimated a reproducibility cloud for our experiments. The boundaries of this cloud are established by averaging the results of the replicates and calculating their standard deviation. Example results can be seen in Fig. 4, where the blue area shows the range of our tests. We can see how drying in the same design tends to follow the same behaviour, despite minor experimental variations. The black line in Fig. 4 describes an additional experiment performed using water as the volatile phase, and which will be discussed later.

To determine how well the exact drying patterns can be reproduced we also made pore-by-pore comparisons of the experiments. For each experiment the image sequence of drying was converted (see Methods) into a pore invasion matrix, *T*_*ij*_, which recorded the time at which each pore, *i*.*e*. at grid coordinates (*i*,*j*), was first recorded as dry. This was used to find which pores had been invaded at any time *t* as

2 $${A}_{ij}(t)=\{\begin{array}{ll}1, & {T}_{ij}\le t\\ 0, & {T}_{ij} > t\end{array}.$$

We confined our attention to the invasion pattern of the main cluster by removing from *A*_*ij*_ all isolated clusters of wet pores. At the same main cluster saturation, *S*_0_, we then compared two invasion patterns *A* and *A*′ by computing their overlap, or match

3 $${\rm{\Delta }}=\frac{{A}_{ij}\cdot {A}_{ij}^{^{\prime} }}{N}$$

where $N={\sum }_{i,j}\,{A}_{ij}$. In other words, we find the fraction of invaded or isolated pores, Δ, that match each other in both patterns, at the same main cluster saturation.

**Figure 5:**
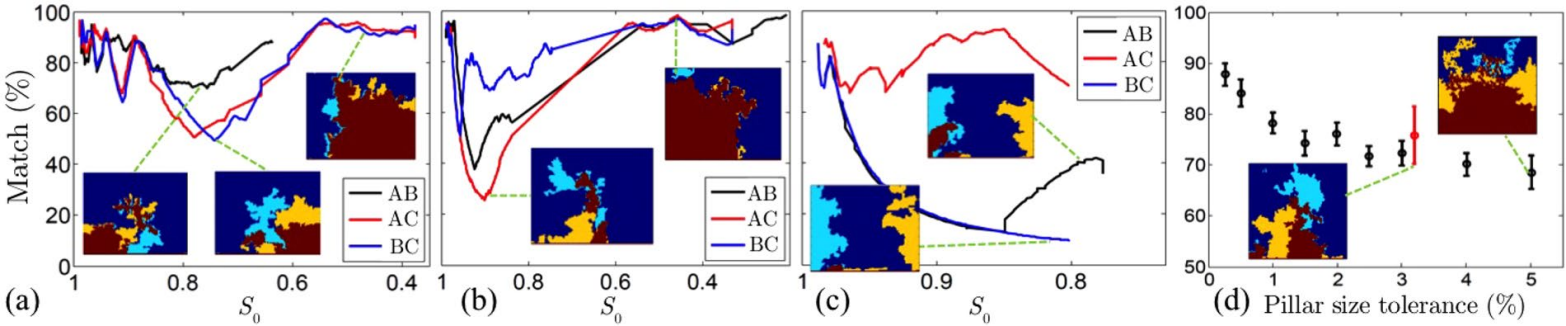
Pore-by-pore comparisons of leading patterns during drying. We made three replicates (A, B, and C) for each of three separate designs. In panels (a–c) we show how well the drying patterns match each other between pairs of replicates. Insets show the invasion patterns. The colours highlight areas that are unsaturated in both samples (red), in one sample only (yellow or light blue) or part of the main cluster in both samples (dark blue). In (**d**) we also show how increasing the manufacturing error in our designs decreases the expected match in the leading patterns of numerical simulations, when *S*_0_ = 0.5. The red point compares this with the average agreement between our experiments and their corresponding simulations.

The evolution of Δ is shown in Fig. 5 for each of our three replicated experiments. Panels a, b show how the invasion patterns in replicates can be reproduced with a match of up to ${\rm{\Delta }}\simeq 90 \%$, and typical values of about 80%. In contrast, Fig. 5c shows a case where the similarity of the patterns is lower than Δ = 10% at breakthrough. However, the insets explain why: drying in this particular sample reached an early choice, based on the near-surface pore sizes, and evolved the drying front either on the left (replicates A, C) or the right (replicate B) side of the cell, hence the lower Δ.

In order to predict the agreement that could be expected between experiments and numerical modelling, we also tested how a random manufacturing error could affect the invasion patterns during drying. For this, we ran a series of numerical simulations with the same initial geometries (and *σ*_*r*_ = 20%), but with random perturbations to the pillar sizes. Figure 5d shows the match that we were able to achieve between different numerical simulations when introducing such a random error. There, we show how increasing the manufacturing error gradually reduces the possible match between patterns, going down to ~70% when as little as a 5% error was introduced. The experimental reproducibility agrees well with that predicted from the model, given our manufacturing tolerance.

Finally, numerical simulations can be more readily undertaken on larger areas than the 100 × 100 grid of our experiments. We repeated a number of observations in numerical simulations of larger cells, of up to 600 × 600 pillars, confirming that there was no significant difference in what one would expect to observe in larger chips. In order to obtain better experimental statistics it may remain worthwhile to design a larger number of pores for some cases. However, based on our experience, it may be difficult to maintain a level enough gap height in microfluidic chips of more than a few square centimetres, for precision applications in porous media flows.

### Disorder and drying behaviour

**Figure 6:**
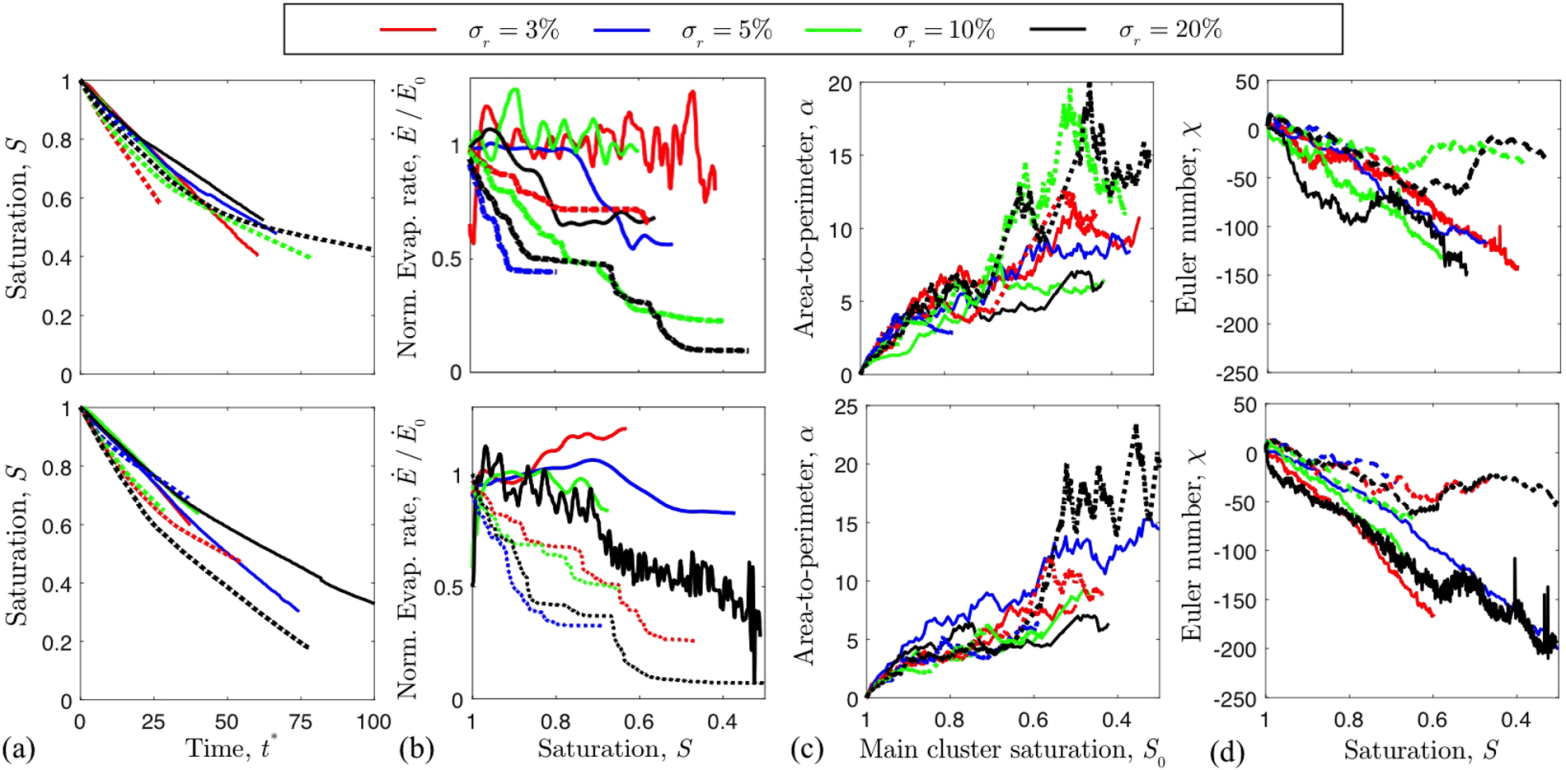
Comparing experimental (solid lines) and simulated (dashed lines) metrics of drying, in matched geometries. The top and bottom rows show results in two different sets of geometries (*i*.*e*. re-randomisations of the pillar sizes, for each given disorder *σ*_*r*_). Panels show (a) how the fluid saturation decreases with time, (b) how the evaporation rate derived from this changes with saturation, (c) how the area-to-perimeter ratio of the leading front increases with main cluster saturation, and (d) how the Euler number decreases with saturation. The apparent oscillations in the drying rate of (b) are due to experimental noise, and the filtering used in calculating the drying rate.

Having characterised the variations expected in both experiments and simulations, we now compare the two cases directly *via* their drying rates and Minkowski functionals. For this, we changed the amount of disorder in our cells, in order to test the effects of heterogeneity on drying. As detailed in the Methods section, we generated distributions of pillars where the pillar radii varied within a 3, 5, 10 or 20% window. Chips with two different randomisations of the pillar radii were made for each level of disorder. Identical geometries were then reproduced in our simulations, and their drying modelled. In Fig. 6a we show the resulting drying curves as a function of the time t*. Then, in Fig. 6b, we look at the relative evaporation rates as a function of the liquid saturation, *S*. In each plot we compare experiments (solid lines) and simulations (dashed lines). Lines of the same colour, within the same plot, refer to matching geometries.

There was no clear effect of disorder on either the time-saturation curves or on how the evaporation rates evolved. However, we observed that experiments would start by keeping a constant rate period and that, in some instances, the beginning of the falling rate period could be seen before breakthrough. In contrast, the simulated evaporation rates always dropped immediately.

In order to understand the origin of this discrepancy we next look at how the invasion patterns changes during drying. This is shown by the Minkowski functionals in Fig. 6c,d. The evolution of the roughness, *α*, of the leading front is reproduced well in the simulations, increasing in a very similar way to that observed experimentally as the main cluster saturation changes. However, the dynamics of the Euler number, *χ*, are not reproduced as well. In the experiments, the receding drying front leaves behind large numbers of clusters that evaporate slowly. In contrast, fewer isolated clusters form in the simulations and these clusters disappear faster than in the corresponding experiments. The difference in the behaviour of isolated clusters could explain the discrepancy observed between the experimental and simulated drying rates. For example, the persistence of the isolated clusters in the experiments effectively maintains higher drying rates by increasing the vapour concentration, and thus enhancing vapour transport, within near-surface pores.

**Figure 7:**
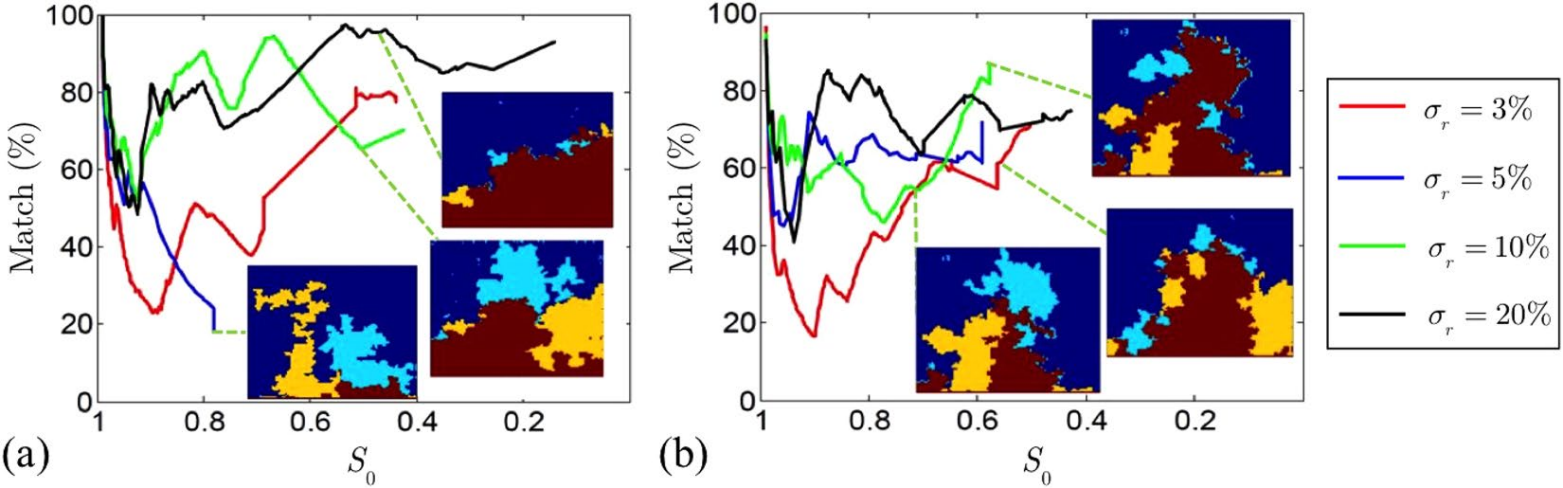
Pore-by-pore comparison of leading patterns, between paired experiments and simulations. The plots show the percentage agreement or match, Δ, between the two cases, versus the main cluster saturation. Panels (a) and (b) show two different randomisations of the pore geometries. The insets show the overlap between the experimental and simulated invasion patterns at particular values of *S*_0_; their colour code matches that in Fig. 5.

**Figure 8:**
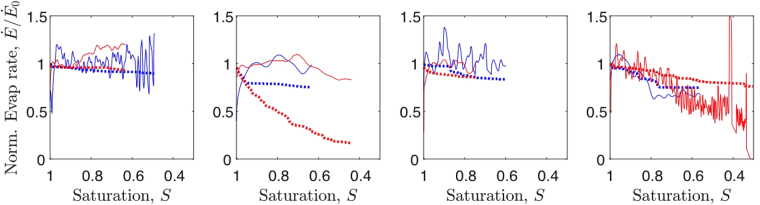
Evaporation rates can be calculated by modelling diffusive evaporation on the experimentally observed sequence of drying patterns, improving their accuracy and recovering the experimental constant-rate period. Experimental rates are shown as solid lines, while simulated rates are in dashes. The poor agreement for one *σ*_*r*_ = 5% case suggesting an isolated fault in that experiment. The apparent fluctuations in experimental rates are the result of filtering noisy evaporation rate data, as in Fig. 6b.

Since the patterns affect the drying rates, we have quantified their agreement more precisely by making pore-by-pore comparisons between experiments and simulations, as we did when comparing experiments in Fig. 5. This result is summarised in Fig. 7. There, we see how the agreement typically stays within, or at least close to, the limit of Δ = 70%. This is a reasonable result when compared to the Δ = 90% threshold at breakthrough we established in the previous section, when comparing pairs of identically prepared experiments. Indeed, in Fig. 5d we show how a manufacturing error of 3%, as measured, brings the best expected agreement into the 70–80% range, as observed. There is one exception where Δ quickly decreases to a value lower than 30%, and does not recover. This chip also showed other significant inconsistencies (see Fig. 8b), suggesting some particular fault, such as a leak, in that test.

In summary, in neither our experiments nor our numerical model was there any significant effect of the magnitude of random disorder in grain size on the invasion patterns. In most cases we showed that the leading patterns are captured well by the model, especially when the pattern agreement is considered in light of the achievable manufacturing precision. However, the model and experiments disagreed on the evolution of the Euler number (or the density of isolated clusters trapped behind the leading front) and the drying rates. These quantities are, in fact, related, as we will now explore.

### Diffusion versus Invasion

The different behaviour of drying rates in experiments and simulations can be explained by considering the different way in which isolated clusters form and behave. In the model, these isolated clusters of liquid evaporate very quickly, whereas they are less active in experiments, often persisting until breakthrough. If we take the experimentally evolved patterns, at various times, we can use our numerical model to predict what the resulting evaporation rate should be, for those exact patterns of wet and dry pores. By doing this for each image in the sequence of a drying experiment, we can test whether the difference between the observed and predicted creation and loss of clusters can explain why the simulated evaporation rates behave differently from the experiments. In the same process, we can also implicitly test whether the simulation accurately models diffusion in the pore space of the experiment. We show the results obtained with this procedure in Fig. 8. This figure shows experimental (solid lines) and simulated (dashed lines) drying rates, where the latter are extracted by taking the experimental pattern sequence recorded in *T*_*ij*_ and using it to estimate the drying rate with our model, based on the resulting vapour concentration gradients. This process clearly improves our estimate, getting experimental and simulated drying rates to agree (excepting again one outlier, in Fig. 8b). The improvement suggests that diffusion and moisture transport are indeed well-modelled in our simulations.

### Wettability

A potential cause for the more limited appearance of isolated liquid clusters in simulations are wettability effects – specifically, non-local cooperative events, where adjacent menisci interact to potentially destabilize each other^43,44^ – that are not considered in the model. To examine such effects, we repeated some experiments with a less wetting fluid, water, which has a contact angle of about 70° on NOA^45^ (as opposed to the $\simeq$3° contact angle of the oil). Using water also slightly changes the viscosity (from 0.77 cSt^37^ to 0.89 cSt), and the evaporation rate of the drying fluid; by performing a sensitivity analysis in our model we confirmed that the change in $\dot{E}$ should be entirely accounted for in our choice of dimensionless parameters. Results from the water-based experiments are shown in Fig. 4, which shows our set of metrics for one particular experiment run three times with oil (blue cloud) and once with water (black line). It can be seen that the fluid saturations, the area-to-perimeter ratios of the leading fronts, and the drying rates behave virtually the same way in both cases. The only observed difference is in the Euler number. Not only is the final amount of clusters lower when using water, but the number of clusters that are formed is always lower at the same saturation. This shows that using a higher liquid surface tension can limit cluster formation.

These observations suggest that a highly wetting fluid, like our oil, can form clusters of isolated pores more readily than a less wetting fluid, like water, during drying. This may be due to the nature of cooperative events, such as menisci becoming unstable by overlapping with each other, which would be more common with a high contact angle^43^. For example, in the related case of fluid invasion, a higher contact angle (of the defending phase) also leads to a more compact invasion front, and less trapped material^43,44^.

## Discussion and Conclusions

The goal of this paper was to rigorously benchmark soft lithography methods for the study of flows in porous media, using a comparative study of drying. To this end we made chips containing an effectively 2-D granular medium with grains of size comparable to those of real soils, improving on both the control of the sample geometry of previous works^5,20^, the pore sizes^18,19^ the throat sizes^15–17^ or the number of objects in the porous medium^21,22^. The was possible *via* standard micro-fabrication techniques, commonly used in microfluidic labs^33–36^, that allow precise control over the positioning of micron-scale objects. Using these methods we also imposed a heterogeneity on the size distribution of the grains and pores, in order to investigate the effects of disorder on drying behaviour in porous media. The microfluidic cells were filled with a volatile oil and allowed to dry. The experiments were then compared to pore-network model (PNM) simulations, of the same geometries. This class of models was chosen due to its ability to combine computational efficiency and accurate descriptions of complex effects^26,27^.

**Figure 9:**
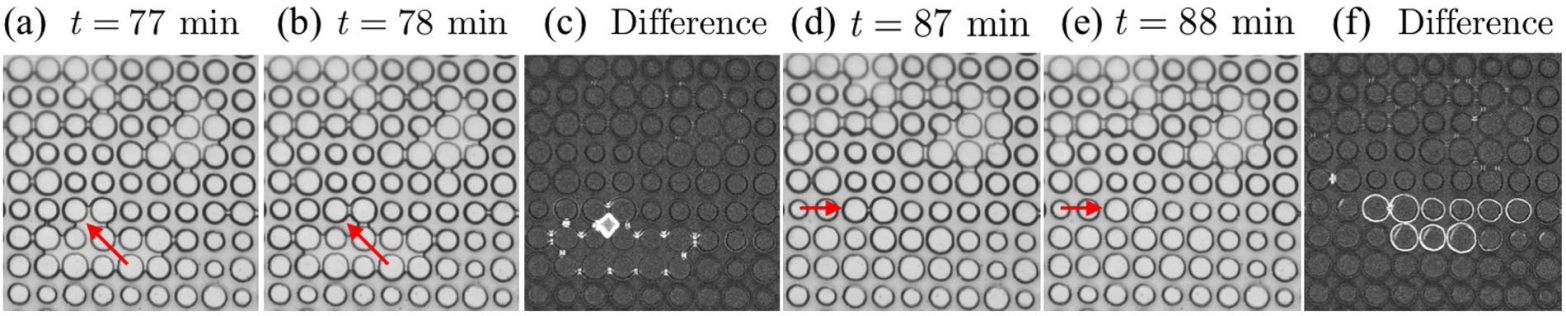
Film flows are limited in our experiments. Between panels (a) and (b) the pore indicated by the red arrow opens. Fluid redistributes throughout the cluster of connected pores, relaxing the menisci around the cluster edges. However, the difference between the two images (**c**) shows that nearby clusters are unaffected by this change. Here, bright regions show the invaded pore, and the menisci that relax around the connected cluster. A few minutes later (**d**) this cluster fully dries. Small rings of liquid remain trapped around several pillars, for example in the row indicated by the arrow. In the next frame (**e**) these rings have disappeared, showing that any film region of drying is quite narrow. Again, (**f**) shows a contrast-enhanced difference image, which highlights the disappearing rings.

The experiments were able to capture the distinction between the constant and falling rate periods of drying. This transition is often not observed in two dimensional drying, as it is known to be strongly affected by the presence, or absence, of either film flows across the upper/lower surfaces of a drying cell, or flows around the corners of such cells^9,13,14,46^. Here, additional visual cues show that film flows are insignificant in our experiments. For example, we could see liquid rings remaining around the pillars for a time after their surrounding pores had drained. Unlike experiments with granular packings^31^, or edged-silicon chips with rougher walls than ours^16^, these rings are not visible significantly behind the trailing trapped fluid clusters, suggesting that any film region in our chips is small, at best. Similarly, microscope observations of the pore-scale dynamics show that adjacent, but isolated, clusters evolve independently from each other. We saw that the throats of menisci in nearby clusters could develop different curvatures, and hence capillary pressures, for example. This suggests, again, that any transport through surface films is limited. Typical examples of cluster and ring observations are shown in Fig. 9. The extended constant-rate period may, instead, be the result of possible corner or gutter flows (*i*.*e*. wetting along the corners, or edges, of the Hele-Shaw cell), and we explore this more fully in our numerically-focussed companion paper^32^.

We have also investigated the agreement between the experimental and simulated drying *patterns*. Specifically, we used the Minkowski functionals as metrics suitable to characterise complex two-phase (here: air-liquid) patterns^41,42^. Their statistical descriptions of the leading patterns were reproduced very well (Fig. 6c), and we have further quantified this match by comparing the patterns pore-by-pore (Fig. 7) at the same main cluster saturation, *S*_0_. We have shown how, given our manufacturing tolerance, the highest expected match in the leading patterns is 70–80%. We do, in fact, reach such a match in most patterns at breakthrough.

However, one noteworthy discrepancy between our experiments and simulations is the number of isolated liquid clusters, or long-lived wet patches in the unsaturated area. A comparison of the evaporation rates of the full pore-network model of drying (see Fig. 6b), with the rates predicted by the model for the experimentally observed drying patterns (Fig. 8), suggests that this discrepancy could also account for the failure of the model to predict our observed drying rates. Investigating the origins of the trapped clusters, we tested the possibility of wettability effects (specifically, cooperative events involving interactions between multiple, adjacent menisci) on drying by repeating experiments with cells filled with water instead of oil. This changed the contact angle of the liquid from ~3° to ~70°. The resulting drying rates and leading patterns were indistinguishable from the corresponding experiment with oil. The only noticeable difference was in the number of clusters formed: water did not break down into as many isolated clusters. However, these isolated clusters still tended to evaporate slower than in the model. This is demonstrated by comparing Fig. 4, in which the Euler number drops throughout drying, with the trend shown by simulations in Fig. 6d, where the Euler number consistently increases in the later stages of drying.

A higher contact angle should further inhibit the formation of fluid films and gutter flows, which may otherwise enhance liquid transport within the porous medium^46^. In the wettability range explored, inhibiting this mechanism only changed the number of clusters formed, but not the evaporation rate or leading pattern of the experiment. The observation of a comparable relative drying rate, despite the different numbers of isolated clusters, suggests that the positions, rather than the density, of clusters predominantly influences the drying rate. This is confirmed by the data in Fig. 8. There, the drying rates were computed by taking an experimental pattern and estimating the drying rate at the next time step. This procedure allows for a much closer estimation of experimental rates by the model, proving the important effect of the pattern of isolated clusters on the drying rate.

Finally, we investigated the effects of adding random heterogeneities to drying porous media. Random disorder does not show any clear effect on the metrics that we have used to characterise our experiments: there is no faster drying rates for higher disorder, for example. Neither did simulations show any noteworthy trends, even though they allow for averaging over many realisations. However, in another paper we investigate the effects of *correlated* disorder, on drying^32^. Using similar experiments and methods to those reported here, there we demonstrate that if there are local patches of larger-than-average or smaller-than-average pores, grouped together over some correlation length, then this additional length-scale of the the heterogeneity in a porous medium can significantly affect drying rates, and drying patterns.

Even though 2D micro-model experiments have been carried out for similar phenomena before^5,15,18,20,44,47^, this is, to our knowledge, the first time that large two-dimensional experiments, with pore-scales of only a few tens of microns, have been performed and directly coupled with PNM. We were able to produce these microfluidic chips with high precision, making such comparisons simpler thanks to the ability of reproducing the same designs in the simulations. These methods can be quite generally applied, and we expect that microfluidic techniques can be used to study the broad class of problems where experimental micro-models have traditionally been applied. This is obviously not limited to drying, but includes fluid-fluid displacement or salt transport and deposition. Such two-dimensional microfluidic micro-models also allow for direct validation of the types of numerical models that are widely used in studying granular packings, saving the time necessary to manufacture the samples and perform simulations by simplifying analysis, and allowing for the removal of unnecessary complications and uncertainties.

## Methods

### Sample design and fabrication

Our experimental chips are thin square cells with a boundary layer that is open to the air along on one edge to allow evaporation, as in Fig. 1. We usually fix this layer to be *λ* = 2 mm wide. However, to test the effects of different boundary layers on drying^10^, it could be varied between 0.5 and 4 mm. On the opposite side of the chip is an inlet that successively splits into eight channels, which are used to fill the cells uniformly with either a perfectly wetting, volatile oil, or water. A 2D porous material is realised by having an extensive array of round pillars in the cell. We can vary the sizes and positions of the pillars to mimic heterogeneous random packings, as would occur in a real soil. Using the soft lithography techniques described below, a minimum feature size of 5 *μ*m and a feature resolution of 2 *μ*m can be achieved. To simplify comparison to numerical modelling we designed our samples as an array of pillars lying on a square grid. Other designs, such as a random close packing or a triangular lattice, could just as easily be manufactured, to match different model geometries^43,44^.

We introduce heterogeneity into the design by choosing the radii of individual pillars from some probability distribution. For example, the radius of each pillar could be randomly selected within the range of 45–55 *μ*m (*i*.*e*. a uniform distribution with a relative width of ±10%). We present here experiments where the pillar radii within any one sample are randomly taken from a uniform distribution with a relative width of *σ*_*r*_ = 3, 5, 10 or 20%. Eight designs are presented, including two different randomisations of each level of disorder. Here we focus on a series of fifteen experiments: each design was run at least once, and three replicate experiments were run for each of three designs, to evaluate the experimental reproducibility. The final experiment involved water as a volatile phase, rather than our usual oil. A set of fourteen additional experiments, with local correlations in pillar size, are studied in a companion paper^32^. Samples have a thickness of 40 *μ*m and contain a 100 × 100 grid of pillars with an average pillar radius of *R* = 50 *μ*m and average throat size of *w* = 25 *μ*m (*i*.*e*. a grid spacing *a* = 125 *μ*m). These designs give square cells of size *L* = 1.25 cm.

The pseudo-2D micro-mechanical models were produced with microfluidic techniques including soft lithography^33,35^. We start by spin-coating a negative photoresist (SU8 3025, MicroChem Corp.) onto a silicon wafer, to obtain a flat layer of thickness *h* = 40 *μ*m. The resist is then exposed to UV light through a mask reproducing the desired pattern. The lit areas crosslink and the unexposed areas are removed by rinsing with a developing solvent (mr-Dev 600, micro resist technology GmbH) and isopropanol, leaving the desired pattern of SU8 on the wafer. We use this raised SU8 structure as a mould, or master, onto which we pour liquid poly(dimethylsiloxane), PDMS, an elastomer that is then cured for one hour in an oven at 75 °C. We peel the cured PDMS layer from the master and use it as a stamp to make a further, negative, PDMS copy of the desired structure. This second mould is then filled with Norland Optical Adhesive 81 (NOA)^34,35^, which will form the body of one of our microfluidic chips. A base for the chip is then prepared by coating an acetate sheet with NOA. We initiate the curing of the two parts of our chip by briefly exposing them to UV light. Once cured, the NOA layer is removed from the PDMS mould. Both components are then placed in a plasma cleaner for one minute. This primes their surfaces so that they can adhere to each other. Finally, the body and base of the chip are pressed together. The base sheet can deform slightly during this step; having such a flexible component in the chip is necessary to guarantee uniform bonding throughout the sample. The bonded chip is then exposed to UV light for 10 minutes, in order to complete curing. Freshly cured NOA is yellow, but fades in intense light. Thus, after fabrication we expose the chip to white light for about 24 hours, in order to stabilise its colour for image analysis. This also stabilises its wettability^45^.

### Setup and image analysis

**Figure 10:**
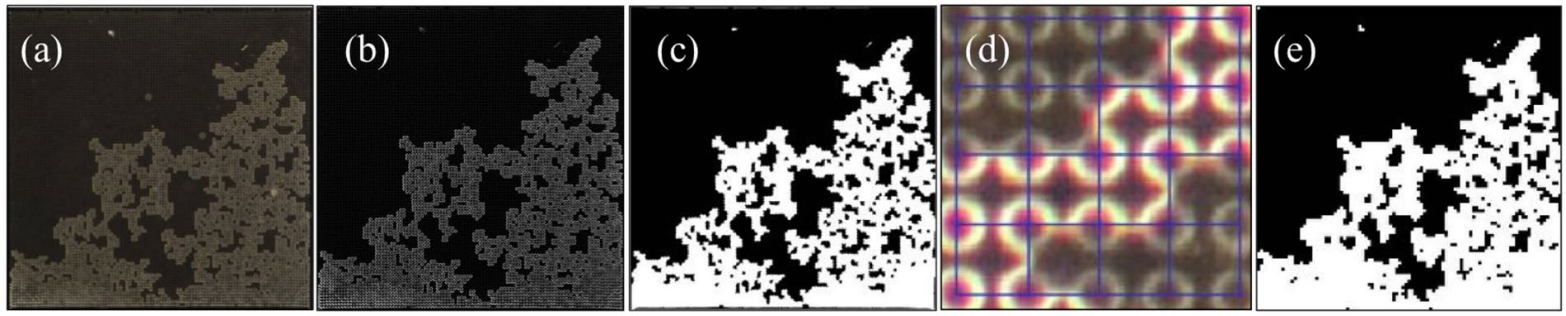
Image analysis. Each raw image (**a**) is flattened into greyscale, filtered and background-corrected to yield (**b**) a cleaned image. This can be thresholded and transformed to remove the fixed pillars and give (**c**) a smooth representation of the drying pattern in black (wet) and white (dry). Alternatively, by looking at (**d**) the image intensity in each pore (here, a 4 × 4 region of pores is highlighted; the bright pores are air-filled), we can construct (**e**) a matrix showing which pores are open (shown as white) or still filled (black).

To observe drying we place a cell filled with a fluorinated oil (Novec 7500, 3M^37^) under a digital SLR camera (Nikon D5100) equipped with a macro lens giving a pixel resolution of 5 *μ*m. The sample is kept horizontal, in order to avoid gravitational effects on drying, and surrounded laterally by a ring of LEDs in an otherwise dark room. The low-angle lighting allows the camera to collect the light mainly scattered from interfaces, in a technique similar to dark-field microscopy. The wet area of the chip thus appears darker than the dry area (Fig. 10a), as the refractive index of the oil is between those of air and NOA. For all experiments the room temperature was fixed to 25 ± 1 °C.

Once in place, the sample dries while the camera images it every minute. The time-lapse image sequence continues until the leading front of the oil-air interface reaches breakthrough, that is, when the drying front first reaches the filling channels, as this disrupts the invasion pattern. The resulting image sequence is then cleaned and processed with Matlab, as demonstrated in Fig. 10. We start by extracting the red colour channel of each image, which contains the best contrast. A bandpass filter is used to remove both the high-frequency noise (cutoff: 3 pixels) and any low frequency variations in intensity (cutoff: 30 pixels). From each image we also subtract the first picture in its sequence, in order to remove constant sources of background noise, such as dust or flaws in the chip. Each cleaned picture (Fig. 10b) is then thresholded to give a binary image that distinguishes between wet (black) and dry (white) areas. Next, we remove any white regions significantly smaller than a pore size, such as wet pillars, in order to leave the wet area completely black. Specifically, we remove objects less than 100 pixels, which can be compared to the average pore size of 310 pixels. We then dilate the remaining white objects, affecting the contour of the dry pillars and the air-liquid interface, and remove the isolated black regions corresponding to pillars in the dry areas. Finally, we erode the picture to reverse the dilation and to give a map (Fig. 10c) of the wet and dry regions where all the pillars have been removed. The accuracy of these image processing steps was monitored, and were occasionally adjusted slightly, to prevent the loss of fine detail in any particular image sequence.

We also employed a second stream of image processing, which summarises the entire image sequence as a discrete matrix, *T*_*ij*_, each entry of which specifies the time at which air first invades the corresponding pore, at location (*i*,*j*). This matrix is extracted from the cleaned time-lapse images described above (Fig. 10b). The pixel coordinates of the pillars at opposite corners of the cell are used to scale the image and map the locations of all the pillars onto a grid. A pore is then defined as the open space between the centres of its adjacent four pillars (Fig. 10d). When looking at the area of the image that is around a single pore, there are four quarter-pillar crowns visible at its corners. We consider a pore dry when these crowns are dry. For any pore all four quarter-pillars were either dry or wet at the same time, as a partially filled pore was neither observed experimentally nor is allowed for in the model. A dry pore shows brighter crowns at the corners than a wet pore, as an air-NOA interface scatters light more than an oil-NOA interface does. We exploit this difference to choose an intensity threshold below which the pore is considered wet. This results in a binary matrix (Fig. 10e) that records the drying state (*i*.*e*. whether each pore is wet or dry) of the chip at each time. By analysing the time-lapse sequence, we can thus determine the first time at which any particular pore (*i*,*j*) is observed to be dry. The pore invasion matrix, *T*_*ij*_, records these times.

### Model

Simulations of drying microfluidic cells are performed using a pore-network model^27,48–50^, based on a minimal set of rules of how pores and fluids interact. The model is sketched below and further details are given elsewhere^32^.

The experimental geometry is discretised into a set of pores, which communicate through throats, as in Fig. 1c. In any simulation the sizes of both types of objects, pores and throats, are specified by the corresponding experimental design. The volume of a pore, defined as the space enclosed by four pillars, is $V=({a}^{2}-{\sum }_{i=1}^{4}\,\pi {r}_{i}^{2}\mathrm{/4})\,h$, where *a* is the grid spacing, *r*_*i*_ is the radius of one of the surrounding pillars and *h* is the thickness of the cell. A throat is the minimal gap between neighbouring pillars and has a rectangular cross-section with a width of *w* = *a* − (*r*_1_ + *r*_2_) and height *h*, if between two pillars of radius *r*_1_ and *r*_2_. Vapour diffusion in the quiescent boundary layer of our chips is modelled by extending our pore-network domain to include additional layers of air “pores”, modelled with pillar radii *r* = 0. This captures the two-dimensional distribution of vapour concentration which develops in the boundary layer as the surface of the porous medium dries^10^.

As diffusion is much slower than pore invasion, we separate these two timescales and model invasion events as occurring instantaneously. In other words, we describe the dynamics of the air-liquid interface as a sequence of steady-state configurations. Between any two sequential invasion events we resolve the equilibrium vapour concentration of the volatile phase, *ρ*, in each air-filled pore (including the boundary layer). Specifically, a mass conservation equation, ${\sum }_{j}\,{J}_{ij}{A}_{ij}=0$, is applied to each open pore *i*, where the summation is done over all neighbouring pores *j*, and *J*_*ij*_ is the vapour mass flux per unit area across a throat of effective cross-sectional area *A*_*ij*_. This flux is approximated by Fick’s first law, *J*_*ij*_ = −*D*(*ρ*_*j*_ − *ρ*_*i*_)/*l*_*ij*_, where *D* is the binary diffusion coefficient of oil vapour in air (*D* = 5 × 10^−6^ m^2^/s). The distance *l*_*ij*_ is the lattice spacing *a*, except where there is an air-liquid interface in the corresponding throat, in which case *l* = *a*/2 (as the air-liquid boundary is half-way between pore centres). The effective area *A*_*ij*_ = *αhw*_*ij*_, where *α* is a geometric correction factor applied to the real minimal area of the throat connecting pores *i* and *j*. A value of *α* = 1.6 was determined for our pore geometry by finite-element simulations of diffusion at the sub-pore scale. In the boundary layer, where there are no constrictions, *α* = 1.

For any given configuration of fluid and air-filled pores, we computed the vapour concentrations and fluxes in the medium by simultaneously solving the system of mass balance equations on all open pores. As boundary conditions for this we assume a vapour saturation (*ρ* = *ρ*_*s*_) along the air-fluid interface, that the vapour density goes to zero (*ρ* = 0) at the open edge of the boundary layer, and that there is no flux across all other edges of the drying cell. The evaporation rate of any connected cluster of fluid-filled pores is taken as the sum of the mass fluxes from across all interfacial throats delimiting that cluster.

We determine invasion of air into liquid-filled pores from the capillary pressure, or equivalently, the meniscus curvature (related *via* the Young-Laplace equation). Each interfacial throat (*e*.*g*. between pillars *i* and *j*) is modelled as a circular constriction with an effective radius of ${r}_{ij}^{\ast }={(\mathrm{1/}h+\mathrm{1/}{w}_{ij})}^{-1}$, and a dynamic meniscus of curvature *C*_*ij*_ trapped in it. The volume of fluid in the throat is related to *C*_*ij*_ by a spherical cap approximation^32^. As evaporation occurs liquid is removed from the fluid-filled clusters, causing their capillary pressure to increase, along with the curvature of the menisci in their interfacial throats. Invasion occurs once the critical curvature for any such throat, ${C}_{ij}^{\ast }=\mathrm{2/}{r}_{ij}^{\ast }$, has been exceeded, ${C}_{ij}\ge {C}_{ij}^{\ast }$. The meniscus then advances through the pore ahead of the critical throat, and the liquid volume released from this pore is redistributed to other interfacial throats in that cluster, relieving their capillary pressure. The new configuration is checked for stability, and either additional pores are then invaded in the same event, or the diffusive balances of the new configuration are recalculated. By alternating between such fast redistribution events, and slow diffusion stages, we model the drying of the porous medium.

### Data availability

The datasets generated and analysed during the current study are available from the corresponding author on reasonable request.
